# The Cerebellum Link to Neuroticism: A Volumetric MRI Association Study in Healthy Volunteers

**DOI:** 10.1371/journal.pone.0037252

**Published:** 2012-05-17

**Authors:** Dennis J. L. G. Schutter, P. Cédric M. P. Koolschijn, Jiska S. Peper, Eveline A. Crone

**Affiliations:** 1 Experimental Psychology, Helmholtz Institute, Utrecht University, Utrecht, The Netherlands; 2 Institute of Psychology, Leiden University, Leiden, The Netherlands; 3 Leiden Institute for Brain and Cognition, Leiden, The Netherlands; Institute of Psychiatry at the Federal University of Rio de Janeiro, Brazil

## Abstract

Prior research suggests an association between reduced cerebellar volumes and symptoms of depression and anxiety in patients with mood disorders. However, whether a smaller volume in itself reflects a neuroanatomical correlate for increased susceptibility to develop mood disorders remains unclear. The aim of the present study was to examine the relationship between cerebellar volume and neurotic personality traits in a non-clinical subject sample. 3T Structural magnetic resonance imaging scans were acquired, and trait depression and anxiety scales of the revised NEO personality inventory were assessed in thirty-eight healthy right-handed volunteers. Results showed that cerebellar volume corrected for total brain volume was inversely associated with depressive and anxiety-related personality traits. Cerebellar gray and white matter contributed equally to the observed associations. Our findings extend earlier clinical observations by showing that cerebellar volume covaries with neurotic personality traits in healthy volunteers. The results may point towards a possible role of the cerebellum in the vulnerability to experience negative affect. In conclusion, cerebellar volumes may constitute a clinico-neuroanatomical correlate for the development of depression- and anxiety-related symptoms.

## Introduction

The idea that the cerebellum may also be involved in cognition and emotion was already examined in the 1970s [1], [2], but it was not until the seminal work published by Schmahmann and Sherman in 1998 that the traditional view of cerebellum involvement being limited to motor-related functions was challenged [3]. Even though mechanistic explanations concerning the role of the cerebellum in non-motor related functions are still hypothetical of nature, it has been proposed that the cerebellum is a structure implicated in timing and monitoring functions [4], and plays a role in the integration of somatic and visceral information [5]. This broader conceptual view is further supported by studies showing reduced emotion regulation capacities following inhibitory repetitive transcranial magnetic stimulation to the cerebellum in healthy volunteers [6]. This finding concurs with patients with cerebellar damage showing impairments in the regulation of behavior [3]. In further support, structural abnormalities of the cerebellum in psychiatric disorders characterized by emotion dysregulation have also been found. In actual fact, volumetric reductions of the cerebellum have been reported for patients with unipolar depressive disorder [7], [8], and patients with anxiety disorder [9]. However, no differences between depressed patients and healthy controls have also been reported [10].

The cerebellum has mono- and disynaptic projections to the limbic system and the frontal cortex respectively, and receives afferent feedback via the pontine nuclei of the brainstem, which offers a neuroanatomical foundation for cerebellar involvement in the regulation of mood [11]. In agreement, a recent structural brain imaging study found an inverse relationship between cerebellar volume corrected for total brain and self-reported depressive mood and anxiety in a community sample of subjects fulfilling diagnostic criteria for post-traumatic stress-syndrome [12]. Interestingly, this study provided evidence for a relationship between early-life traumatic experience and cerebellar volume in adulthood. Despite this relation it remains unclear whether reductions of cerebellar volumes may in fact constitute a structural neuroanatomical vulnerability factor for the development of mood disorders. To address this issue a 3T structural magnetic resonance imaging (MRI) association study was conducted to test the hypothesis that cerebellar volume inversely correlates with neurotic personality traits in healthy volunteers.

## Materials and Methods

### Participants

Thirty-eight healthy male (n = 18) and female (n = 20) right-handed volunteers, mean age ± SD, 21±2 years, were recruited by local advertisement. None of the volunteers had a history of psychiatric or neurological conditions and participants were screened for MRI contraindications. One male participant indicated the use of recreational drugs and was excluded from the study. History of alcohol consumption (in years) and tobacco smoking was inquired to examine the possible influence of alcohol and smoking on cerebellar volumes [13], [14], but see [15]. Participants were naïve to the aim of the study and paid for participation. Written informed consent was obtained from all participants. The study was approved by the local ethical review board of Leiden University, Leiden, the Netherlands, and carried out in accordance with the standards set by the Declaration of Helsinki (Seoul Amendments).

### Personality assessment

The personality traits related to anxiety and depression were rated with the Dutch version of the two corresponding 8-item subscales of the revised NEO Personality Inventory (NEO-PI-R) [16], [17]. The subscale anxiety indexes personality characteristics associated with the experience of nervousness, tension, fear and worry. The subscale depression indexes personality characteristics related to the susceptibility of individuals to experience feelings of guilt, sadness, hopelessness and loneliness. Both subscales correlate to neuroticism, emotional instability and subjective feelings of inadequacy [17], and associated with increased risk of developing mood disorders such as depression and anxiety [18].

### MRI acquisition and preprocessing

Scanning was performed on a 3T Achieva whole body scanner (Philips, Best, The Netherlands) at Leiden University Medical Center. A high-resolution 3D T1-FFE scan was obtained (TR = 9.760 ms; TE = 4.59 ms, flip angle = 8 degrees, 140 slices, 0.875×0.875×1.2 mm^3^ voxels, FOV = 224×168×177 mm^3^) with a total scan duration of 296 s. All T1 scans were reviewed and cleared by a radiologist. No anomalous findings were reported. MRI scans were individually checked on motion artifacts or other sources of signal loss.

**Figure 1:**
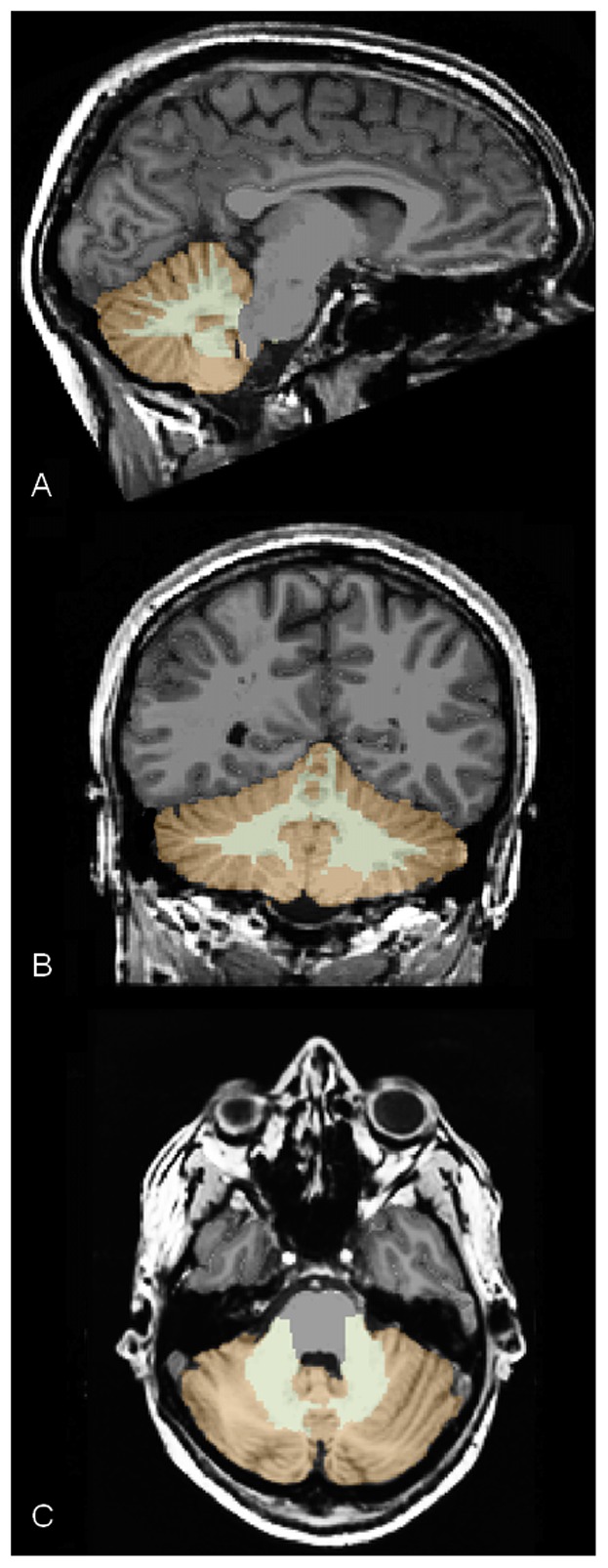
Representative example of sagittal (A), coronal (B), and axial (C) slice of the cerebellum from a participant as segmented by FreeSurfer. Color legend: brown – cerebellum gray matter; light brown – cerebellum white matter.

Total brain and cerebellar gray and white matter volumes were measured automatically using the software FreeSurfer version 5.0 (http://surfer.nmr.mgh.harvard.edu) [19]–[21]. Briefly, processing consisted of removal of non-brain tissue [22], and automatic segmentation of (sub)cortical gray matter structures [23]. This procedure automatically assigns a neuroanatomical label to each voxel in an MRI volume, based on probabilistic information automatically estimated from a manually labeled training set. First, the image is rigid body registered to a probabilistic brain atlas followed by non-linear morphing to the atlas. Manually segmented images were previously used to create statistics about how likely a particular label is at any given location in the brain. This serves as a Bayesian prior for estimating the label of a given voxel in a given patient's brain image based on the maximum a posteriori probability. The segmentation uses three pieces of information to disambiguate labels: (1) the prior probability of a given tissue class occurring at a specific atlas location, (2) the likelihood of the image intensity given that tissue class, and (3) the probability of the local spatial configuration of labels given the tissue class. The technique has previously been shown to be comparable in accuracy to manual labeling [23]. Finally, all cerebellar segmentations were visually inspected for accuracy prior to inclusion in the group analysis. Figure 1 depicts an example of a representative anatomical T1-weighted scan that was used for automated volume extraction.

### Statistical analyses

Normality of the data was checked by running the Kolmogorov Smirnov (KS) test and through inspection of the kurtosis and skewness of the distribution. Data were considered to be normally distributed when the KS test was not significant and the kurtosis and skewness values fell between −1 and +1. Depending on the outcome of these analyses parametric Pearson's product-moment correlations or non-parametric Spearman's rank correlations were performed to test our primary hypothesis of an inverse association between total brain corrected cerebellar volume and neurotic personality traits. The influence of total brain volume and history of alcohol consumption was examined through partial correlations. The possible influence of smoking (n = 6) was examined by comparing cerebellum volumes and neurotic personality traits with non-smokers (n = 29) using a Mann-Whitney U test for two independent samples. The alpha level of significance was set at 0.05 (two-tailed). In case of significant associations between cerebellar volume and depression and anxiety scores, the contributions of total gray and white matter were explored in a second series of correlations. Finally, a statistical difference test between dependent correlations was performed.

## Results

Data of two male participants were discarded due to excessive head movements during scanning. For the remaining thirty-five participants, the depression (mean ± SD, 25.3±6.1) and anxiety scores (mean ± SD, 25.3±6.7) were comparable to the depression (mean ± SD, 23.7±5.4) and anxiety scores (mean ± SD, 24.5±5.5) scores found in the general population (n = 1305) [17]. The KS tests did not yield significant effects on the volumetric and personality variables (all *p*-values>0.35). Kurtosis and skewness values of the variables ranged between −0.85 and 0.52. These results indicate that the data followed a normal distribution and Pearson's product-moment correlations were performed.

**Figure 2:**
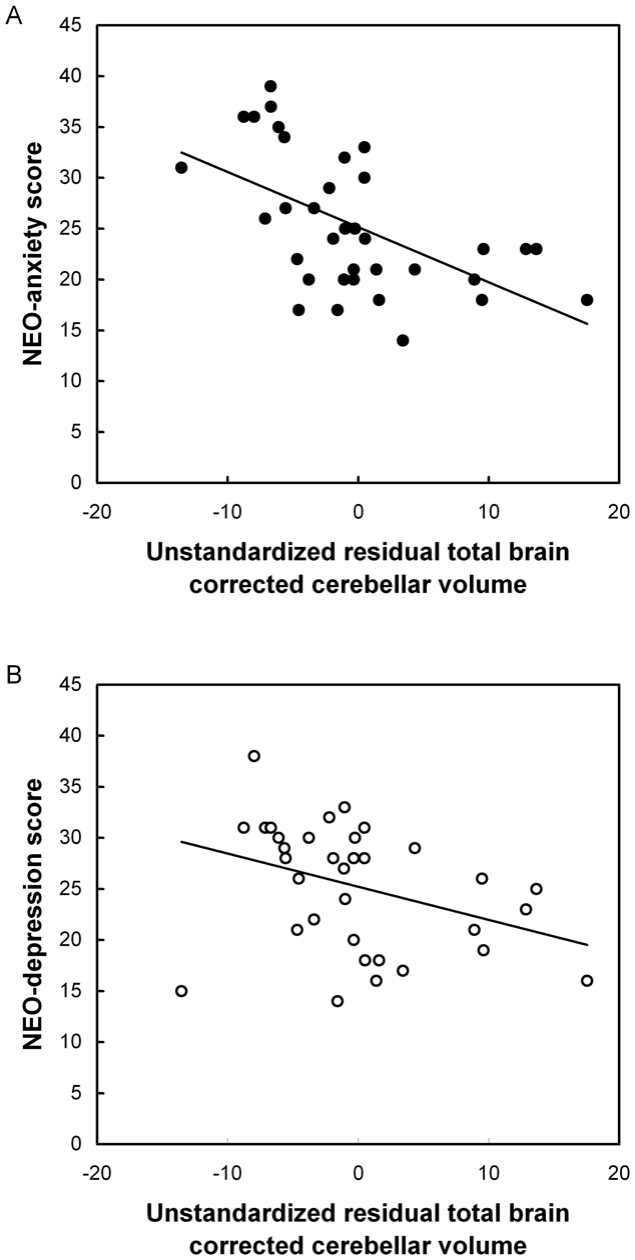
Unstandardized residuals of cerebellum volume corrected for total brain volume are inversely associated with the NEO-PI-R anxiety (A) and depression score in healthy volunteers (B).

The anticipated inverse correlations between total cerebellar volume (mean ± SD, 125.3±11.3) and anxiety score, r(33) = −0.35, *p* = 0.04, and between total cerebellum volume and depression score, r(33) = −0.40, *p* = 0.02, were found. After correction for total brain volume correlations remained significant between total cerebellar volume and anxiety score, r(32) = −0.56, *p* = 0.001, and depression score, r(32) = −0.38, *p* = 0.03. Notably, partialling out years of alcohol consumption did not affect the presently observed correlations (corrected r-values>0.37, *p*-values<0.03). No differences were found between smokers and non-smokers on cerebellar volume, Z = −0.70, *p* = 0.51, depression scores, Z = −0.68, *p* = 0.5, and anxiety scores, Z = −0.99, *p* = 0.33. Figure 2 shows the scatter plots of the significant associations between total brain corrected cerebellar volume and anxiety score (A) and between total brain corrected cerebellar volume and depression score (B) are shown. The additional exploratory correlations revealed significant associations between cerebellar gray matter volume (mean ± SD, 91.3±9.3) and anxiety, r(33) = −0.32, *p* = 0.06, and depression, r(33) = −0.35, *p*<0.04. For cerebellar white matter volume (mean ± SD, 34.0±4.1) the association with anxiety score was not significant, r(33) = −0.23, *p* = 0.19, whereas the association with depression score reached borderline significance, r(33) = −0.32, *p* = 0.06. Finally, the statistical difference tests for dependent correlations of the gray and white matter correlation coefficients did not demonstrate significant differences between gray and white matter contributions (t-values<0.60, *p*-values>0.59).

## Discussion

The aim of this study was to test the hypothesis that smaller cerebellar volumes may constitute a vulnerability factor for the development of mood disorders. In line with our expectations, results showed that cerebellar volume was inversely related to depression and anxiety scores in healthy volunteers, demonstrating, for the first time, a neuroanatomical linkage between cerebellar volume and neurotic personality traits in a non-clinical sample. No differences between gray and white matter volumes in explaining the volumetric relation with depression and anxiety were found, indicating that both gray and white matter contribute equally in explaining the relation between cerebellar volume and neurotic personality traits. However, it should be noted that demonstrating an absence of a statistical difference between gray and white matter does not indefinitely prove that no distinctions between gray and white matter exist.

Our findings extend earlier results of clinical studies that found associations between cerebellar volumes and depression- and anxiety-related disorders. Also, our data complement functional neuroimaging studies showing changes in cerebellar blood flow during the experience of negative mood states [24], by adding a structural correlate to the link between cerebellum and personality traits. Furthermore, increasing evidence points towards a topographic organization of cerebellar functions. The vermis is proposed to be more directly coupled to affective processes, whereas the posterior cerebellar hemispheres are more involved in processes associated with cognitive behavior. On the notion that the experience of depression and anxiety can be considered a phenomenon particularly associated with impaired regulation of negative affect, then one would expect stronger associations with the posterior lobules of the cerebellar hemispheres. From this viewpoint, neurotic personality traits may be associated with the aberrant cerebellar modulation of cognitive loops in the cerebral cortex. Moreover, neuroimaging studies have shown activation of midline structures including the vermis in response to the experience of negative affect [25], suggestive for a relation more closely linked to the vermal part of the cerebellum. Given the fact that the experience of depression and anxiety involves complex interactions between cognitive and affective processes is it not unlikely that the cerebellum as a whole is involved. In sum, on the basis of the present findings further studies on the functional decomposition of the cerebellar processes underlying depression and anxiety seem worthwhile.

Depression and anxiety scores have been suggested to reflect the most salient aspects of the neurotic personality [17] and often correlate, as in this study (r = 0.64, *p*<0.001), which suggests that both traits share common grounds in explaining the neurotic personality type. We speculate that the association between cerebellar volumes and neuroticism may be more closely tied to the valence dimension of mood states as subjective arousal levels are generally high during the experience of anxiety and low during the experience of depressive mood. However, the extent to which the current relation is confined to neurotic personality traits remains to be determined.

There is recent evidence showing that cerebellar volumes are also smaller in patients with bipolar disorder [26], but see [27], which suggests that the cerebellum may serve a more general function with respect to the regulation of emotions and mood states. A reason for the inconsistencies concerning cerebellar abnormalities reported across studies may in part be due to the fact that earlier studies used computed tomography and low resolution magnetic resonance imaging to determine cerebellar volumes. These imaging techniques may have been less sensitive to detect volumetric differences or associations between patients and controls. The view that the cerebellum subserves a general regulatory function fits with the proposed idea of the universal cerebellar transform function in which the cerebellum supposedly acts as a comparator of actions and desired outcome to promote internal homeostasis [28]. These actions do not need to exclusively relate to information in the motor domain, but can involve information associated with the cognitive and affective domain as well [25]. Impairments in the modulatory function of the cerebellum may result in the loss of coordination of cognitive and affective information processing streams (i.e., the dysmetria of thought hypothesis), giving rise to phenomenological symptoms of depression and anxiety as a consequence of lower emotion regulation capacities [29]. From this viewpoint, negative mood states can be conceptualized as phenomenological manifestations of disturbed internal homeostasis resulting from certain stressors that initiate action to restore equilibrium [28]. Additional evidence for cerebellar involvement in the processing of negatively valenced emotional stimuli was recently provided by a study in which cerebellar function was manipulated with transcranial direct current stimulation [30].

Moreover, it has been shown that there exists a positive relationship between neuroticism and high levels of the stress-related hormone cortisol [31]. In addition to the findings that high levels of glucocorticoids are associated with depression and anxiety, volumetric reductions of brain structures containing high density glucocorticoid receptors including the cerebellum in response to chronic stress have been reported as well [32], [33]. Alternatively, it is possible that a reduction in cerebellar volume is a secondary effect of the increased cortisol levels. This would imply that the observed interrelations between cerebellar volume and symptoms of depression and anxiety are in fact epiphenomenal associations. However, the monosynaptic efferent connection of the cerebellum to the hypothalamus [34], together with the high corticoid receptor density in the cerebellum [32] may provide a possible physiological feedback mechanism by which the cerebellum regulates hypothalamic activity. In spite of the fact that cortisol levels were not available and the link between cerebellar volumes and negative mood is correlational of nature, our results are nonetheless in agreement with our predictions derived from prior clinical studies of an association between cerebellum and neurotic personality traits [6]–[9]. Furthermore, the fact that glucocorticoids may negatively impact cerebellar volumes provides a possible corollary for the tendency to experience depression and anxiety. On a more speculative account, the inverse association in healthy volunteers may thus indicate a possible clinico-neuroanatomical substrate for the susceptibility to develop depressive and/or anxiety related disorders. This interpretation would agree with notions of cerebellar involvement in negative affect and impairments in the regulation of emotion following cerebellar damage and the effects of glucocorticoids on cerebellar volume. Future longitudinal studies are however needed to address the issue of cerebellar volume contributing to the develoment of mood disturbances. Finally, even though the present findings lend further support to the proposed link between the cerebellum and non-motor related functions, at this point the mechanistic insights that could explain this association remain circumstantial and hypothetical of nature.

In conclusion, the present structural MRI study shows that cerebellar volume covaries with neurotic personality traits in healthy volunteers and may point to a possible role of the cerebellum in the vulnerability to experience negative affect and develop mood disorders.
